# The relationship between maternal vitamin D status during third trimester of pregnancy and maternal and neonatal outcomes: A longitudinal study

**DOI:** 10.18502/ijrm.v17i1.3818

**Published:** 2019-03-07

**Authors:** Mahboobeh Shakeri, Sima Jafarirad

**Affiliations:** ^1^Student Research Committee, Ahvaz Jundishapur University of Medical Sciences, Ahvaz, Iran.; ^2^Nutrition and Metabolic Diseases Research Center, Ahvaz Jundishapur University of Medical Sciences, Ahvaz, Iran.; ^3^Department of Nutrition, School of Para-medicine, Ahvaz Jundishapur University of Medical Sciences, Ahvaz, Iran.

**Keywords:** *Vitamin D*, *Pregnancy*, * Infant*, * Growth*, * Delivery*, * Blood glucose.*

## Abstract

**Background:**

Vitamin D deficiency is a common nutritional concern and leads to several problems among some population groups.

**Objective:**

The aim of the current study was to evaluate the relationship between maternal vitamin D status and gestational weight gain, maternal biochemical parameters, mode of delivery, and infants' growth indices at birth.

**Materials and Methods:**

A longitudinal study between March and June 2017 was carried on 82 mothers in Ahvaz. Blood samples of each mother were obtained at the mean of the third trimester to assay lipid indices (total cholesterol, triglycerides, low-density lipoprotein, and high-density lipoprotein cholesterol), fasting blood sugar, and 25-hydroxy vitamin D. Anthropometric assessment of newborns was recorded from neonatal health card at birth.

**Results:**

Mean maternal 25-hydroxy vitamin D level was 22.52 nmol/L; 7.33% of mothers had vitamin D deficiency, 76.6% had vitamin D insufficiency, and 15.9% were normal. The mean neonate birth weight, length, and head circumference of mothers who were on the third tercile of 25-hydroxy vitamin D serum level was significantly higher than those in the first tercile (p < 0.001, p = 0.004 and p < 0.001, respectively). Maternal vitamin D serum level had an adverse relationship with fasting blood sugar.

**Conclusion:**

Low levels of serum vitamin D may cause adverse pregnancy outcomes and delivery of infants with insufficient growth at birth.

## 1. Introduction 

Vitamin D is a key nutritional factor for maternal and infants' health. The fetus level of vitamin D is totally dependent on their mother's levels of 25-hydroxy vitamin D (25(OH)-D). The circulating levels of 25(OH)-D is the marker to represent vitamin D status. It correlates with the intake of vitamin or sun exposure during pregnancy (1). Vitamin D has a key role in fetal growth by regulating the parathyroid hormone and increasing calcium absorption. The complication of low consumption of calcium status or insufficient vitamin D is high secretion of parathyroid hormone (2).

Vitamin D deficiency is common in pregnant women which affects not only the mothers' health but also of the infants (3). The deficiency probably causes a weak growth of fetal during pregnancy. Some studies have emphasized on post- and past-delivery effects of vitamin D on weak bone mineralization of infants that has an important relationship with short growth age (4).

Low maternal vitamin D level has been linked with preeclampsia, gestational diabetes, postpartum depression, and stillbirth (5). Furthermore, vitamin D insufficiency in uterus or early stages of life increases the risk of several diseases such as childhood wheezing, respiratory infection, and type 1 diabetes mellitus (6). So normal vitamin D level is necessary during pregnancy, and recognition of risk factors and primary diagnosis of vitamin D status during pregnancy is essential to prevent deficiency and its complications (4).

Mothers' weight can affect vitamin D serum levels. One study showed that the mean serum level of 25(OH)-D in pregnant women with pre-pregnancy overweight [body mass index (BMI)=25-29.9] was more than that of pregnant women with normal pre-pregnancy BMI (BMI=18.5-24.9) (7), while other studies confirmed that obese mothers had low levels of 25(OH)-D (8).

Ahvaz is a city located in the south west of Iran with mostly sunny days, but it seems that in Iran, the clothing style and lack of seasonal foods can affect vitamin D status in women. This study was done to evaluate the vitamin D status among pregnant women residing in this city. Since there were few studies about the linkage of maternal vitamin D serum level with pre-pregnancy BMI, gestational weight gain, maternal blood sugars, lipid profiles as well as anthropometric measurements of neonates, the present study was aimed to find the association between maternal vitamin D status during third trimester and gestational weight gain, maternal blood sugar, lipid profiles, mode of delivery, and infant's weight, length, and head circumference at birth.

## 2. Materials and Methods

### Subjects' recruitment

This study was a longitudinal and cross-sectional survey and conducted on pregnant women living in Ahvaz (a city in the south west of Iran) between March and June 2017. Subjects recruited were healthy pregnant women referred to two heath centers' representatives of the East and the West of Ahvaz. The inclusion criteria included age range 18–45 yr at the expected date of delivery and women who were during the third trimester of pregnancy. Those having chronic infectious diseases, metabolic disorders, gestational diabetes, gestational hypertension, preeclampsia, eclampsia, twin pregnancy, eating disorders (anorexia, bulimia), emotional disorders (depression, stress, and anxiety), drug consumption, and taking multivitamin supplements (out of health centers routine programs) were excluded. Applying the inclusion and exclusion criteria, 88 pregnant women were selected.

### Data gathering 

A demographic questionnaire including personal information (age, education, job, ethnic). In addition, disease history was investigated. Gestational weight gain was assessed from the data of the pregnant women recorded files. The trimesters were defined as first (less than 14 completed wk), second (14–27 completed wk), and third (28 completed wk until delivery). Weight gain during the third trimester was estimated using the difference between the first and last weight recorded divided by the number of weeks between the two visits (9). Three days food recall (two work days and one holiday) questionnaire was completed for all subjects to determine micro- and macro-nutriments intake.

Weight and height of mothers were determined with an accuracy of 0.1 kg and 0.1 cm, respectively. BMI was calculated using this formula: bodyweight**/**(height)${}^{2}$; in this formula bodyweight is presented as kilogram and height as meter. An appropriate weight gain was recommended by the institute of Medicine and National Research Council guideline based on pre-pregnancy BMI. Those in the underweight (BMI<18.5) category were recommended to aim at gaining 12.5–18 kg, normal (BMI=18.5–24.9) 11.5–16 kg, overweight (BMI=25–29.9) 7–11.5 kg, and obese (BMI>30) 5–9 kg (9).

For measuring vitamin D, 5 ml of venous blood were obtained from each mother at the mean of third trimester to assay 25(OH)-D level using the enzyme-linked immunosorbent assay (ELISA, EUROIMMUN, Germany). Tercile of serum vitamin D were determined to evaluate the mothers' vitamin D status.

Fasting blood sugar (FBS) and lipid profiles (triglyceride, low-density lipoprotein-cholesterol (LDL-C), high-density lipoprotein-cholesterol (HDL-C), and total cholesterol were determined using an auto-analyzer (Hitachi 911, Japan).

After delivery, the infant's growth indices at birth (including weight, height, and head circumference) were collected from their health cases in health centers.

### Ethical consideration

The study was approved by the Ethics Committee of the Ahvaz Jundishapur University of Medical Sciences (IR.AJUMS.REC.1395.837). All subjects completed an informed consent form.

### Statistical analysis

Statistical analysis was performed using SPSS software (Statistical Package for the Social Sciences, version 17.0, SPSS Inc. Chicago, USA). Data were expressed as means ± standard deviations (SD) for continuous variables or number (percentage) for categorical variables. One-way analysis of variance (ANOVA) was used to analyze the difference of each variable among mothers with different vitamin D status. Tukey post-hoc analysis was done to confirm the significant difference of variables among groups of mothers with different vitamin D status. Odds ratio (OR) was determined to measure if vitamin D insufficiency could predict the probability of low weight, length, and head circumference at birth. p-value < 0.05 was considered as significant level.

## 3. Results

From 88 pregnant women, 82 subjects delivered healthy and term neonates; hence, 82 women were included in the analysis. The characteristics of these women are described in Table I.

### Demographic data 

53.6% of women were between 18–30 yr and 46.3% women were>30 yr. According to the BMI levels before pregnancy, mothers were categorized as underweight: (2.4%), normal weight: (56.1%), overweight: (31.7%), and obese: (9.8%). The number in each group for total weight gain that was categorized as insufficient, adequate, and excessive were 15(18.3%), 39(47.6%), and 28(34.1%) women, respectively. None of the subjects followed a special diet.

### Maternal 25-hydroxy vitamin D serum level

Of all mothers, 7.33% had vitamin D deficiency, 76.6% insufficiency, and 15.9% were in normal range. Also, mothers were categorized based on the tercile of 25(OH)-D serum levels to first, second, and third tercile.

Analysis revealed that the birth weight of infants who were born by mothers in the first tercile of 25(OH)-D serum level was significantly lower than that of the second and third tercile (p < 0.001). Moreover, women in the first tercile had shorter infants compared to women in the third tercile of vitamin D serum levels (p = 0.004) (Table II). The OR confirmed that vitamin D deficiency and insufficiency could predict 1.3 and 1.32 times lower birth weight (LBW) and lower infants' length at birth, respectively (Table III). ANOVA indicated significant difference between maternal vitamin D status and neonates' head circumference (Table II). Also, vitamin D deficiency and insufficiency could predict 1.29 times infants with head circumference (HC) under 25th percentile (Table III).

**Figure 1:**
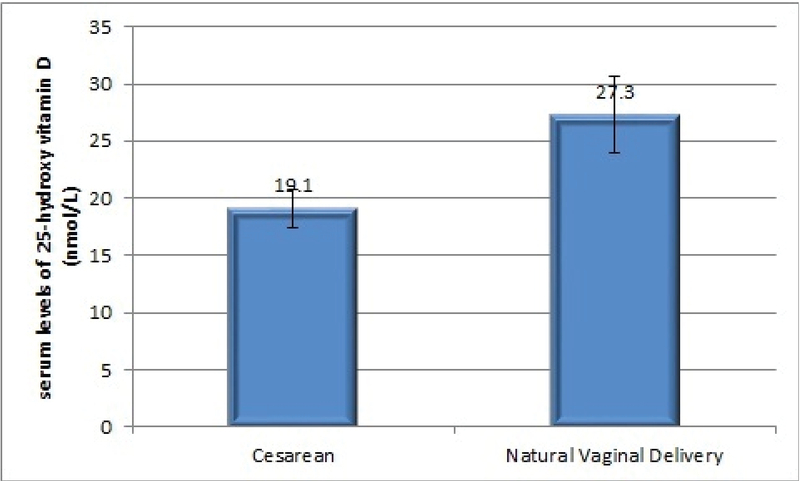
The relationship between vitamin D status of pregnant women and mode of delivery (Chi-square test).

Data also revealed that mothers who delivered babies via cesarean sections had significantly less vitamin D serum level compared to mothers who experienced natural vaginal delivery (p = 0.027) (Figure 1).

There was no relationship between maternal vitamin D status and gestational weight gain and serum lipid profiles (total cholesterol, triglyceride, LDL-C, and HDL-C), but mothers in the first tercile of vitamin D status had more FBS than those in the third tercile; however, it was in the normal range (Table II).

**Table 1:** General data of pregnant subjects.


Variable (*N* = 82)		
Maternal age (yr)*		29.8 ± 5.45
Total weight gain (kg)*		13.80 ± 4.22
Maternal vitamin D serum level (nmol/L)*		22.52 ± 15.99
Birth weight (g)*		2990 ± 430
Height (cm)*		48 ± 3.57
Head circumference (cm)*		34.05 ± 1.13
Fasting blood sugar (mg/dL)*		76.51 ± 8.02
Total cholesterol (mg/dL)*		238.93 ± 43.93
High density lipoprotein (mg/dL)*		58.97 ± 10.59
Low density lipoprotein (mg/dL)*		138.70 ± 35.00
Triglyceride (mg/dL)*		203.04 ± 70.66
Education**		
	Diploma and lower education	43 (52.4)
	Academic education	39 (47.6)
Ethnicity **		
	Turk and Kurd	3 (3.65)
	Lor	23 (28)
	Persian	12 (14.6)
	Arab	44 (53.6)
Note: * Data presented as mean ± SD. ** Data presented as *n* (%).		

**Table 2:** The differences between maternal weight gain and biochemical parameters during pregnancy and neonates' anthropometric indices at birth among mothers in different tercile groups of 25-hydroxy vitamin D serum level.


	**Maternal Vitamin D Status**			p-value*
	**First tercile**	**Second tercile**	**Third tercile**	
Neonate anthropometric measurements				
Birth weight (g)	2200 (121)${}^{a}$	2970 (351)${}^{b}$	3491 (220)${}^{b}$	< 0.001
Birth length (cm)	43.75 (0.88)${}^{a}$	47.63 (2.41)${}^{ab}$	51.76 (5.53)${}^{b}$	0.004
Birth head circumference (cm)	32.41 (0.37)${}^{a}$	34.01 (1.07)${}^{b}$	35.00 (0.54)${}^{b}$	< 0.001
Maternal weight gain				
Total weight gain	14.83 (4.70)	13.91 (4.30)	12.76 (3.73)	0.56
Biochemical parameters				
FBS (mg/dL)	78.37 (9.10)${}^{a}$	78.98 (8.46)${}^{ab}$	72.35 (4.09)${}^{b}$	0.002
T.CHO (mg/dL)	241.85 (45.48)	244.25 (44.18)	231.00 (42.83)	0.34
HDL (mg/dL)	56.25 (10.39)	59.92 (9.39)	60.67 (11.67)	0.5
LDL (mg/dL)	142.48 (38.18)	140.33 (32.92)	133.55 (34.40)	0.11
TG (mg/dL)	203.73 (70.45)	215.74 (81.23)	190.17 (59.27)	0.26
Note: Data presented as mean (SD); Different letters confirm significant differences (Tukey post-hoc analysis); *One way analysis of variances;				
FBS: Fasting Blood Sugar; T.CHO: Total cholesterol; HDL: High Density Lipoprotein;				
LDL: Low Density Lipoprotein; TG: Triglyceride.				

**Table 3:** The OR for low neonate growth indices at birth according to vitamin D status of mothers.


**Neonate growth indices at birth**		**Maternal Vitamin D status**				
	**Deficient or insufficient**	**Sufficient**	p-value*	**OR**	**95% CI**
Birth weight (Percentile)	< 25	26 (100)	0	0.007	1.302	1.128–1.504
	> 25	43 (76.8)	13 (23.26)		
Birth length (Percentile)	< 25	29 (100)	0	0.003	1.325	0.136–1.545
	> 25	40 (75.5)	13 (24.5)		
Birth head circumference (Percentile)	< 25	25 (100)	0	0.008	1.295	1.125–1.492
	> 25	44 (72.2)	13 (28.8)		
Note: Data presented as *n* (%); OR: odds ratio; CI: confidence interval; *Chi-square test.						

## 4. Discussion

The present study was performed on pregnant women to find the relationship of maternal vitamin D serum level with weight gain during pregnancy and infants' weight, length, and HC at birth.

### Maternal vitamin D status and infant's growth indices at birth

A significant association was found between maternal lower vitamin D serum levels and LBW, which was compatible with the results of other studies (10–13). One study in Netherlands and another from Australia found significantly LBW among infants born to women with vitamin D deficiency (12, 13). Also Bodnar and colleagues have reported a positive relationship between vitamin D deficiency and neonatal LBW in white mothers and not in black mothers (11). However, other researchers found no association between maternal vitamin D status and normal- or low birth weight (14).

Our finding indicated a significant positive relationship between maternal vitamin D serum levels and neonatal length, which is in line with the previous research that showed a significant relationship between inadequate vitamin D intake during pregnancy and low neonatal birth weight and shorter infant height (15, 16), while other studies did not find any relationship between maternal vitamin D deficiency and neonatal length (10).

Also, in this study, mothers with vitamin D deficiency or insufficiency delivered neonates with HC ≤ 33 cm, consistent to another study (10). However, inconsistent to our results, another study showed an independent correlation between neonatal HC and maternal vitamin D level (17).

It seems that the effect of vitamin D on growth indices at birth is related to the role of this vitamin in bone mineralization and the following low levels of it lead to less bone growth (4). Consequently, the levels of mothers' serum vitamin D may predict the infants' growth indices at birth.

### Maternal vitamin D status and serum biochemical parameters

Our finding showed that serum levels of 25(OH)-D had significantly inverse correlation with FBS that is in line with another study (18). Also, our results support previously reported results in which serum concentrations of 25(OH)-D levels during 24–28 wks of gestation were significantly lower in women with gestational diabetes mellitus than in groups with normal levels of vitamin D (19). While our findings found no significant relationship between mothers' vitamin D status and lipid indices (total cholesterol, triglycerides, LDL-C, and HDL-C), another survey that studied 25(OH)-D serum levels among a population of men showed a significant association between low levels of 25(OH)-D and high levels of LDL-C, but no association with HDL-C (20). However, in a study that was performed on Saudi pregnant women, serum vitamin D in the subjects surprisingly correlated positively with the serum levels of triglycerides and cholesterol (21).

Some proposed mechanisms could display the relation between vitamin D deficiency and risk of elevated blood glucose or gestational diabetes mellitus. Protective effects of vitamin D on pancreas beta-cells finally lead to the regulation of insulin secretion (22). Also, some evidence related to vitamin D deficiency with abnormal glucose and dyslipidemia and other epidemiologic studies has revealed that pregnant women with gestational diabetes are more likely to be vitamin D-deficient mothers (19).

### Maternal vitamin D status and mode of delivery

In the present study, a significant difference was found between the mode of delivery and mothers' vitamin D status, which confirms the results of a cross-sectional survey and a clinical trial that both of them show the vitamin D effect on the risk of primary caesarian-section (23, 24). In that cross-sectional study, 25(OH)-D levels<37.5 nmol/L were associated with four-fold increased odds of requiring first-time (primary) cesarean section (23); while the result of that randomized clinical trial showed no effect of vitamin D supplementation on the mode of delivery (24). Some reasons could be pointed out to describe this association such as the key role of vitamin D in calcium homeostasis (including an effect on muscle function and may help to starting labor); although, further exploration would be required (25).

The limitation of the study was that it did not evaluate the effect of supplements intake and the amount of sunlight exposure on the vitamin D status. We speculate that these factors should be considered in future studies.

## 5. Conclusion

This study shows that maternal low 25(OH)-D level could be related to their infant's lower weight, shorter height, and lower HC at birth. Also, it was associated with the mode of delivery. However, serum vitamin D in these subjects did not correlate with the serum levels of triglycerides, cholesterol, LDL-C, and HDL-C, but a significant adverse association was found between FBS and maternal vitamin D levels. So, the findings of this study showed that vitamin D during pregnancy may have ample importance and it can be suggested that vitamin D status screening during pregnancy could help prevent low infant growth indices at birth and undesirable effects on the mothers.

##  Conflict of interest 

The authors declare that they have no conflicts of interest.
